# The Re-Opening of the School of Pharmacy

**Published:** 1911-10-07

**Authors:** 


### The Re-opening of the School of Pharmacy.

In opening the seventieth session of the School of Pharmacy on Wednesday, October 4, Dr. J. Macdonald Brown, who delivered the inaugural address, correctly explained the causes why pharmacy, as a calling, was so poorly paid that a whole series of accessories had had to find a place on the shelves of chemists' shops, so that the pharmacist might earn a living. Underselling, and the unfair competition engendered by drug-stores, general stores, and the like; the many detrimental aspects of the "drug-tablet" question, which in very essence removes the whole sphere of administration of remedies from doctor and chemist alike; the credulity of the British public with regard to patent medicines—all these tend to divert pharmacy from the scientific path and make it a less profitable calling than it was a quarter of a century ago. Dr. Brown gave some useful words of advice to the students. Examinations, he said, ought to be regarded as milestones along the road of knowledge, and should not be looked upon as goals which, once reached, mean the end of studious work. The ideal life of the student is one in which regular, conscientious daily work should be thoroughly done from the beginning of the session to its close. The idler, who moans away his earlier months, often strives, by burning the midnight oil, to get himself into trim against the awful examination-day, and though he may be successful then, the knowledge he has gained is not sure and lasting, and is easily dissipated. At the close of the meeting the Hanbury gold medal, which is awarded biennially for high excellence in the prosecution or promotion of original research in the chemistry and natural history of drugs, was presented to M. Eugène Leger, a distinguished French pharmacist, who has done a large amount of research work in connection with the chemistry of the active constituents of drugs. M. Leger, by the way, is chief pharmacist to the Hôpital Saint-Louis, Paris.

